# Supplementary material for: Manipulation of fractionalized charge in the metastable topologically entangled state of a doped Wigner crystal
Source: Nat Commun. 2023 Dec 11;14:8214. doi: 10.1038/s41467-023-43800-3 (PMC10713626; doi:10.1038/s41467-023-43800-3)
Supplement: Supplementary file 1 — Supplementary Information [file 41467_2023_43800_MOESM1_ESM.pdf]

Supplementary information

Manipulation of fractionalized charge in the metastable topologically entangled state of a doped Wigner crystal

Anze Mraz*[1,2], Michele Diego[1], Andrej Kranjec[1], Jaka Vodeb[1], Peter Karpov[3], Yaroslav Gerasimenko[1,4], Jan Ravnik[1], Yevhenii Vaskivskyi[1,5], Rok Venturini[1,5], Viktor Kabanov[1], Benjamin Lipovšek[2], Marko Topič[2], Igor Vaskivskyi[1,4] and Dragan Mihailovic[1,4,5]

[1] *Jozef Stefan Institute, Dept. of Complex Matter, Jamova 39, SI-1000 Ljubljana, Slovenia*

[2] *Faculty for Electrical Engineering, University of Ljubljana, Tržaška 25, SI-1000 Ljubljana, Slovenia*

[3] *Arnold Sommerfeld Center for Theoretical Physics, Ludwig Maximilian University, München, Germany*

[4] *CENN Nanocenter, Jamova 39, SI-1000 Ljubljana, Slovenia*

[5] *Faculty for Mathematics and Physics, University of Ljubljana, Jadranska 19, SI-1000 Ljubljana, Slovenia*

email: anze.mraz@ijs.si

Supplementary Note 1. DESCRIPTION OF THE PHASE DIAGRAM OF 1T-TaS_2.

Under thermodynamic conditions the material undergoes a series of first-order phase transitions: from a metal to an incommensurate (*IC*) charge-density-wave (CDW) state[1] at 550K, then to a nearly commensurate (*NC*) state at 350K, which may be described as a regular patchwork of commensurate CDW domains, separated by discommensurations[2] and eventually an insulating commensurate (*C*) state below ~180 K (Supplementary Fig. 1a, slow cooling)[3] that is unique amongst TMDs. On heating, a triclinic, stripe-like electronic phase is observed between 220 and 280 K[4], before the system reverts to the *NC* state (Supplementary Fig. 1a, slow warming).

In response to photoexcitation or charge injection through an electrode, the insulating *C* state breaks apart either into a so-called 'hidden' (*H*) metastable metallic domain state[5–7] (Supplementary Fig. 1b), or an amorphous Wigner glass state[8], depending on excitation conditions. Subsequent heating from the *H* state to room temperature first sees a relaxation to the *C* state at approx. 70 K[9] and then follows the same steps as in the equilibrium phase diagram (Supplementary Fig. 1a). Resistance-temperature (R-T) curves corresponding to the phase diagram in Supplementary Fig. 1b are presented in Supplementary Fig. 1c; cooling to the *C* state (black line), switching from *C* to the *H* state (dashed blue line) and heating from the *H* state back to room temperature (red line). Insets to Supplementary Fig. 1c are scanning tunnelling microscope (STM) images of the *C* (black arrow) and *H* state (red arrow).

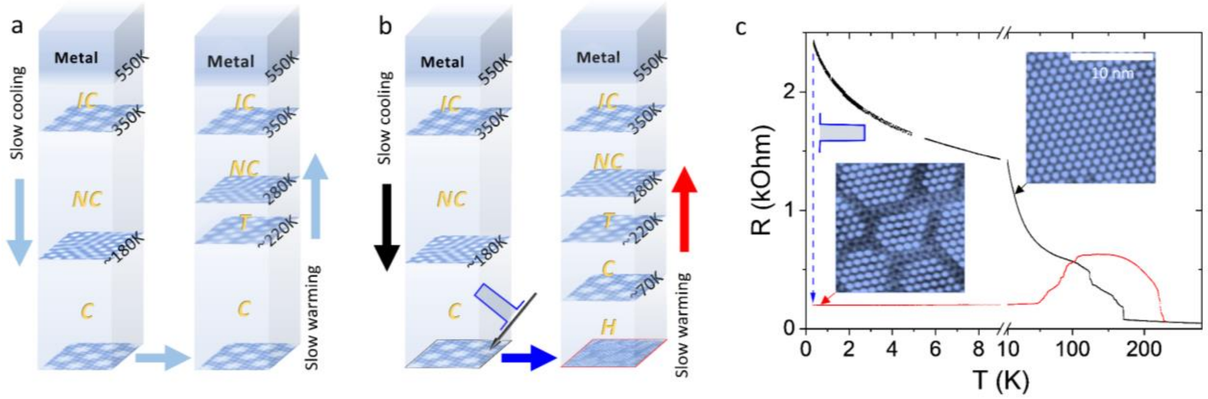

Supplementary Figure 1: a) Equilibrium phase diagram. b) Non-equilibrium phase diagram with excitation to the *C* state and switching to the *H* state at low temperature. c) R-T curve of cooling to the *C* state (black line), switching from *C* to the *H* state with a current pulse (blue dashed line) and heating from the *H* (red line) in a nanofabricated device with electrodes 100 nm apart. The inserted images correspond to STM measurements in the *C* (black arrow) and the *H* (red arrow) state respectively.

Supplementary Note 2. FULL 'ERASE' SEQUENCE CAPTURED WITH THE STM

In Supplementary Fig. 2 we present the full set of STM images obtained during the 'erase' sequence that was presented in the main manuscript in Fig. 2. Each of the images shown was captured after each incremental increase of the 'erase' current pulse and only a select few were presented in Fig. 2c and later analysed in Fig. 3. In the top left corner of each image is the value of the 50 μs long current pulse sent between tips #1 and #2, after which the STM image was recorded with tip #3 (Fig. 2a). All of the STM images were taken on the same spot as can be seen by the defects in the images, which are also highlighted in the next chapter. The same sequence as seen in Supplementary Fig. 2 is also presented in a video Supplementary Movie 1 showing the same evolution of the domain structure. We also note that the area which is imaged with the STM (~40x40 nm^2) represents only a very tiny part of the switched area between the outer STM tips that are ~2.7 μm apart (illustrated in Supplementary Fig. 3). If circular current path shape between the outer tips is assumed, we can estimate that we are observing only ~0.03% of the entire switched area on the surface.

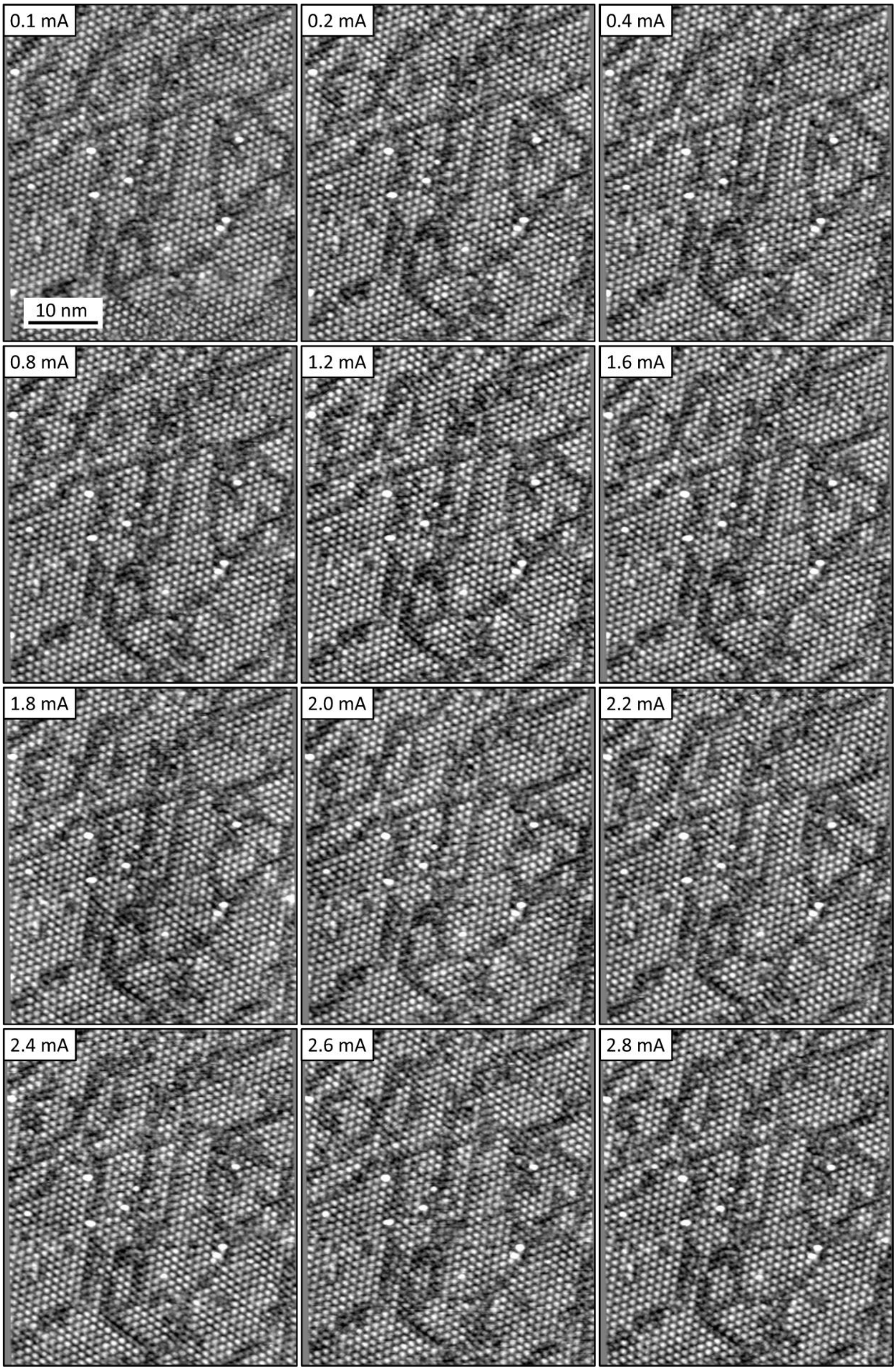
0.1 mA
10 nm
0.2 mA
0.4 mA
0.8 mA
1.2 mA
1.6 mA
1.8 mA
2.0 mA
2.2 mA
2.4 mA
2.6 mA
2.8 mA

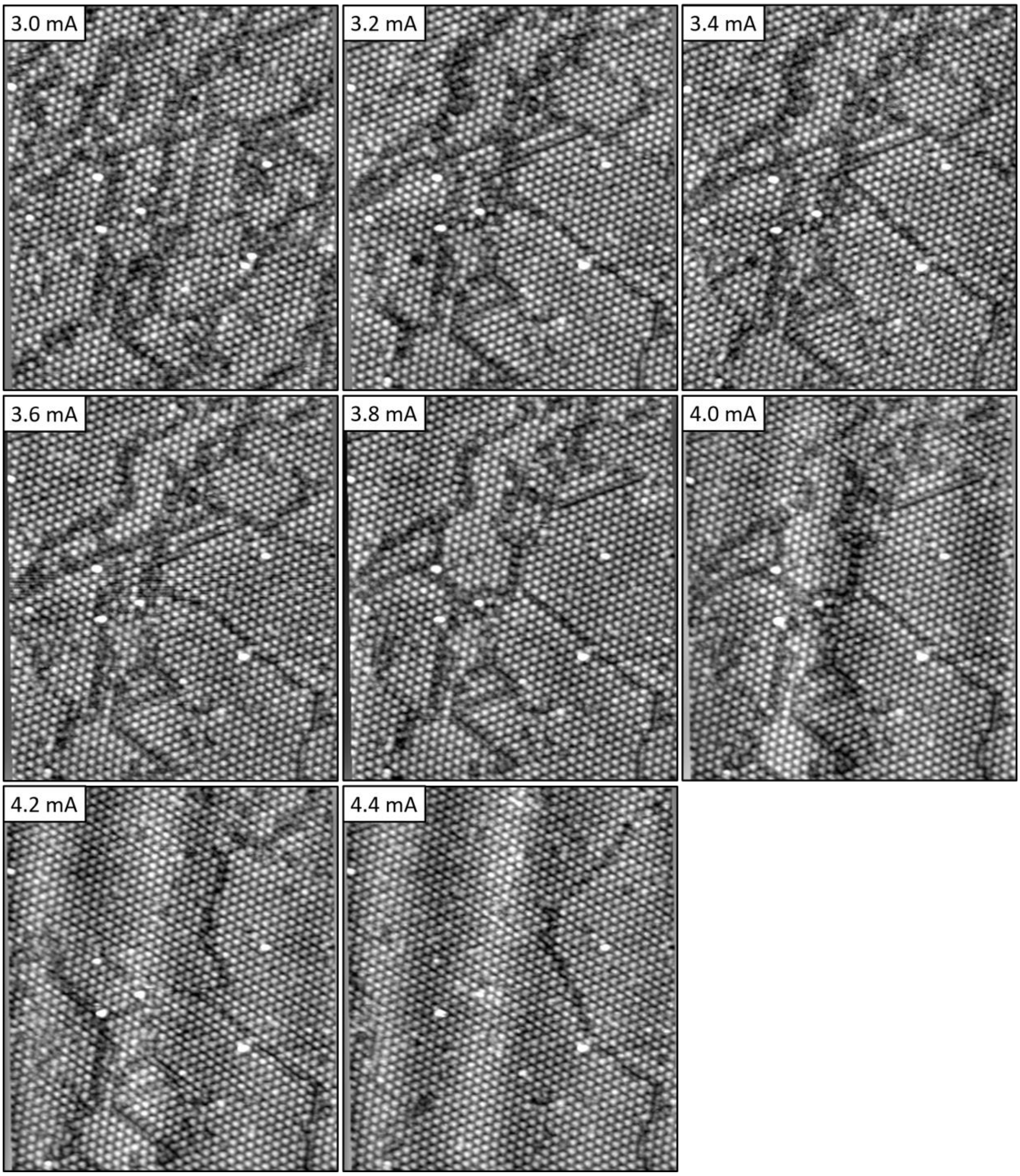

Supplementary Figure 2: Full set of STM images of the 'erase' sequence in Fig. 2 in the main manuscript. Each image shows a recording of the same area with STM tip #3 after each current pulse, incrementally increasing in amplitude from 0.1 mA up to 4.4 mA. The bias voltage between the tip and the sample used for scanning was -0.8 V, and the tunnelling current was 1 nA. All images are corrected for the drift of the STM.

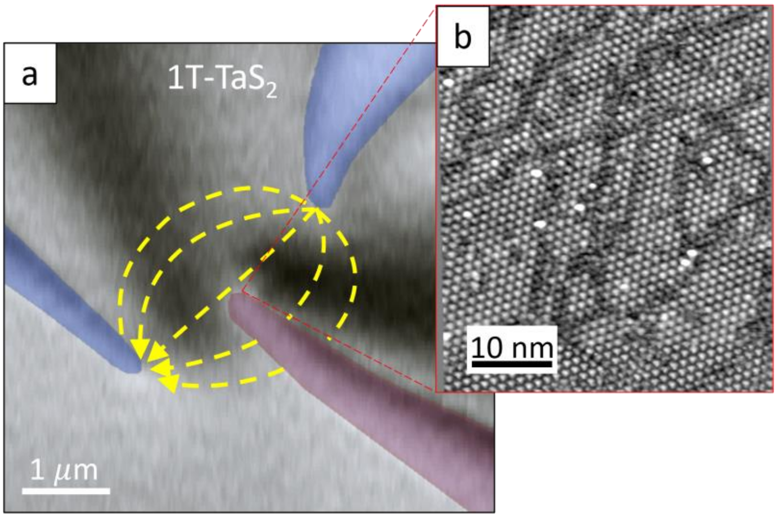

Supplementary Figure 3: a) *In-situ* SEM image of the STM setup where outer tips (blue) are used to supply electrical current pulses and the middle tip is used to scan the surface, as shown in b). Yellow arrows illustrate the path of electrical current. b) STM scan of the surface. Note the scale bar difference between a) and b).

Supplementary Note 3. DIFFERENT 'WRITE' AND 'ERASE' EVENTS RESULT IN DIFFERENT CHARGE CONFIGURATION PATTERNS

Supplementary Fig. 4 shows the continuation of switching immediately after the 'erase' sequence presented in the main manuscript and in Supplementary Fig. 2 above, and demonstrates different configuration pattern of the domain wall network between different switching events. Supplementary Fig. 4a-c correspond to the first and last two panels of Supplementary Fig. 2, respectively, with the added highlighting of the defects that indicate constant STM scanning position on the sample. Following Supplementary Fig. 4c is Supplementary Fig. 4d, which is an 'erased' state reached through a series of six 50 μs long pulses with 4.0 mA amplitude, after which the sample was 'written' again with a single 50 μs long 7 mA current pulse resulting in the domain wall pattern seen in Supplementary Fig. 4e. Even though there seems to be some directionality in the orientation of the domain walls, which corresponds to the position of the current source contact tips #1 and #2, the microscopic pattern of the domain wall configurations changes between different 'write' and 'erase' events, meaning that lattice imperfections do not appear to play a significant role in the formation of a specific pattern.

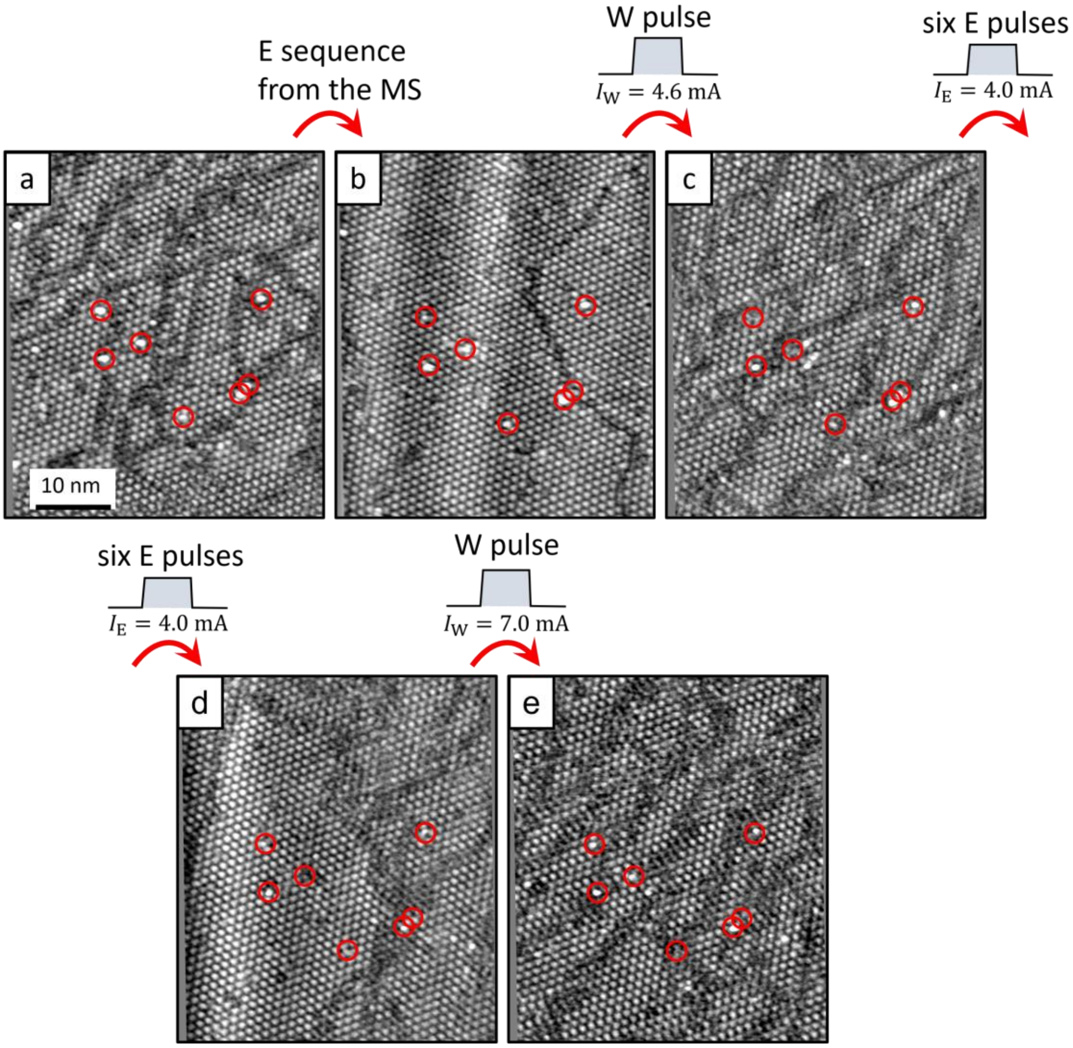

Supplementary Figure 4: STM images with a few marked defects in the domain wall state (red circles) indicating the same position of scanning between images. a) The first panel from Supplementary Fig. 2, showing H state. b) The end of the 'erase' sequence from Supplementary Fig. 2 at 4.4 mA pulse amplitude. c) 'Re-written' state with a pulse of 4.6 mA amplitude. d) 'Erased' state reached from c) by applying a series of six pulses with 4.0 mA amplitude. e) another 're-written' state reached from d) by applying a single 7 mA current pulse.

Supplementary Note 4. REPEATED 'ERASE' SEQUENCE ON A DIFFERENT SAMPLE CAPTURED WITH THE STM

To show reproducibility we repeated the same experiment on a different 1T-TaS_2 sample, which shows very similar behaviour to the one presented in the main manuscript. The STM setup at 4 K with three STM tips can be seen in an *in-situ* SEM image in Supplementary Fig. 5a, where current carrying tips #1 and #2 are positioned ~4.5 µm apart and advanced ~50 nm into the 1T-TaS_2 material, and tip #3 is positioned somewhere in the middle and used to scan the surface of the material. Supplementary Fig. 5b and c show an STM recording of the initial insulating ground *C* state captured with tip #3 and the switched metallic hidden *H* state, respectively. The *H* state was reached after applying a 50 µs long electrical current pulse of amplitude 16.9 mA. Red circles in Supplementary Fig. 5b and c indicate either deformed polarons or voids in the polaronic lattice (probably due to imperfections in the crystal lattice), which can be used to register the position of scanning in both images. We note that some defects in the

polaronic lattice in Supplementary Fig. 5b become domain wall crossings or knots in the switched state in Supplementary Fig. 5c.

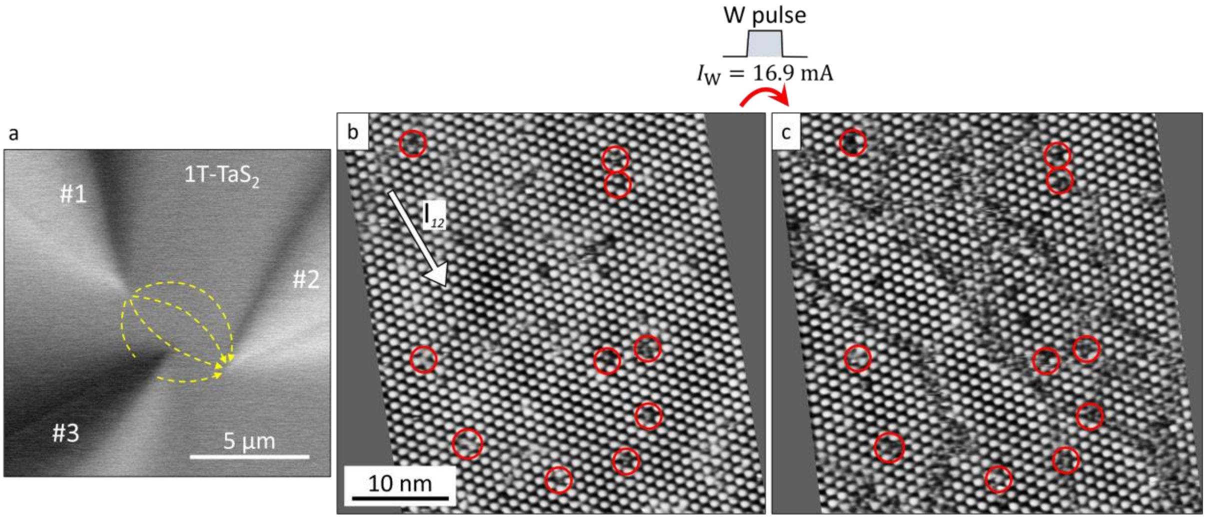

Supplementary Figure 5: STM setup of the repeated experiment. a) SEM image of the experimental setup on 1T-TaS_2. STM tips #1 and #2 are used to supply electrical current pulses laterally through the sample. The current path is schematically shown by yellow arrows. Tip #3 is used to scan the area in between the outer tips. b) STM image of the initial *C* state with some defects in the polaronic lattice marked by red circles. c) STM image of the switched *H* state reached with a 50 µs long current pulse of 1 with the same positions of defects marked. We observe the same scanning area on the sample between the two images.

To 'erase' the *H* state (Supplementary Fig. 5c and Supplementary Fig. 6, *H* state) back to the *C* state we again employ the 'erase' sequence with incrementally increasing 50 µs long current pulses while capturing STM images of the domain wall network after each pulse, which is presented in Supplementary Figs. 6 to 10. In the top left corner of each panel on each figure is the value of the current pulse sent between tips #1 and #2, after which the STM image was recorded with tip #3. The most substantial change in the charge configuration landscape can be observed at the current value 13.0 mA (Supplementary Fig. 9), which then continues to evolve slightly until the sample is 're-written' at the current value 17.0 mA (Supplementary Fig. 10). We can notice that the domain wall motif which is formed after the 're-write' (Supplementary Fig. 10, 17.0 mA) is different to the original pattern (Supplementary Fig. 6, *H* state), showing again that any lattice imperfections most likely do not substantially affect the formation of a specific domain wall formation. What could be important to the arrangement of the charge configuration on the top surface is the charge configuration mosaic in the bottom layers, which can be discerned in the last few panels in Supplementary Fig. 10, and as was illustrated in Fig. 4r and s in the main manuscript. The same 'erase' sequence as in Supplementary Figs. 6 to 10 can also be observed in a video Supplementary Movie 2.

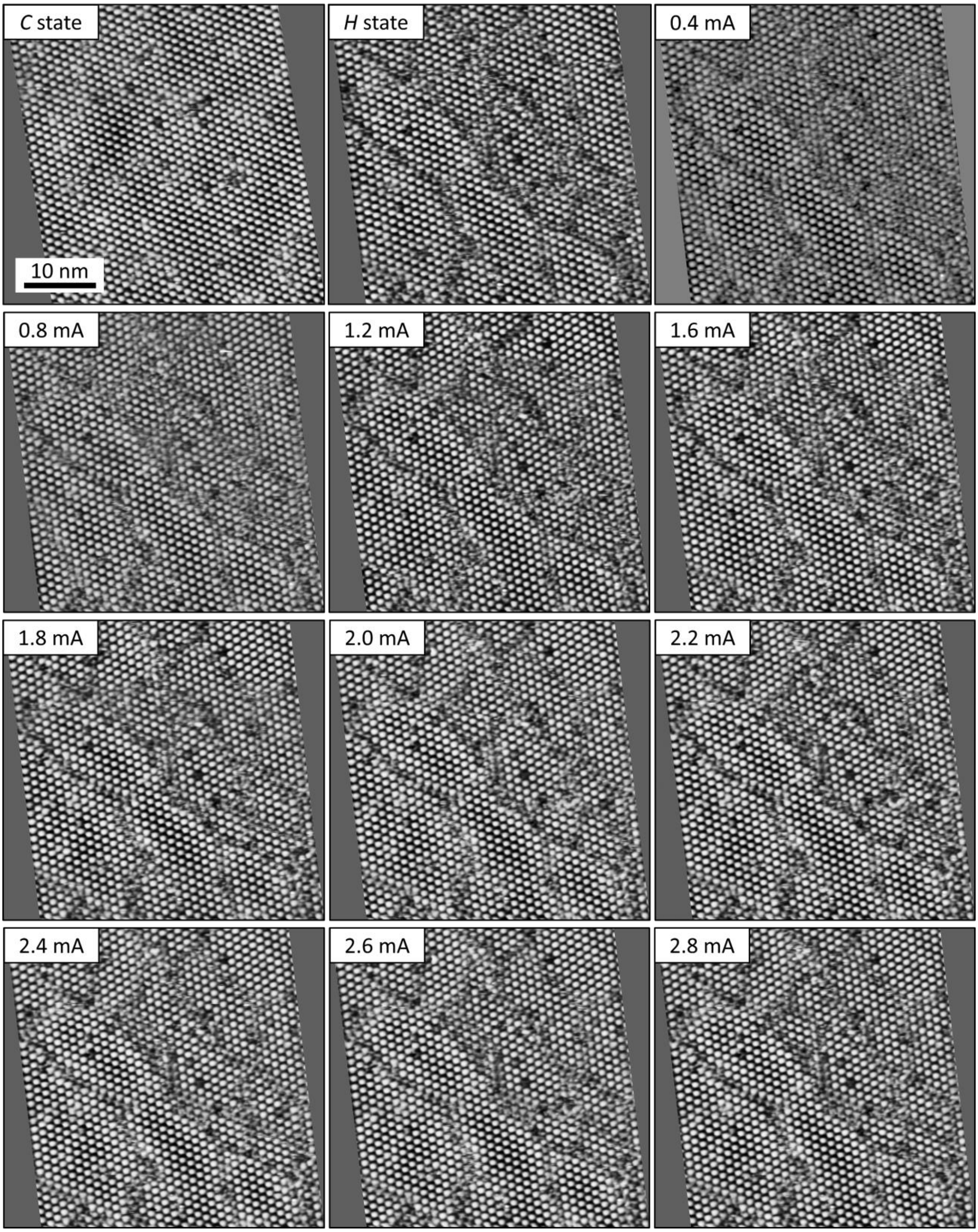

Supplementary Figure 6: Repeated 'erase' sequence on a different 1T-TaS_2 sample at 4 K. First two panels show the insulating ground *C* state and the switched metallic *H* state with measured resistance values, respectively. Following is the 'erase' sequence recorded with an STM after each current pulse up to 17 mA, as indicated in the top left corner of each panel. The bias voltage between the tip and the sample used for scanning was -0.8 V, and the tunnelling current was 50 pA. All images are corrected for STM drift as stated in the Methods section in the main manuscript.

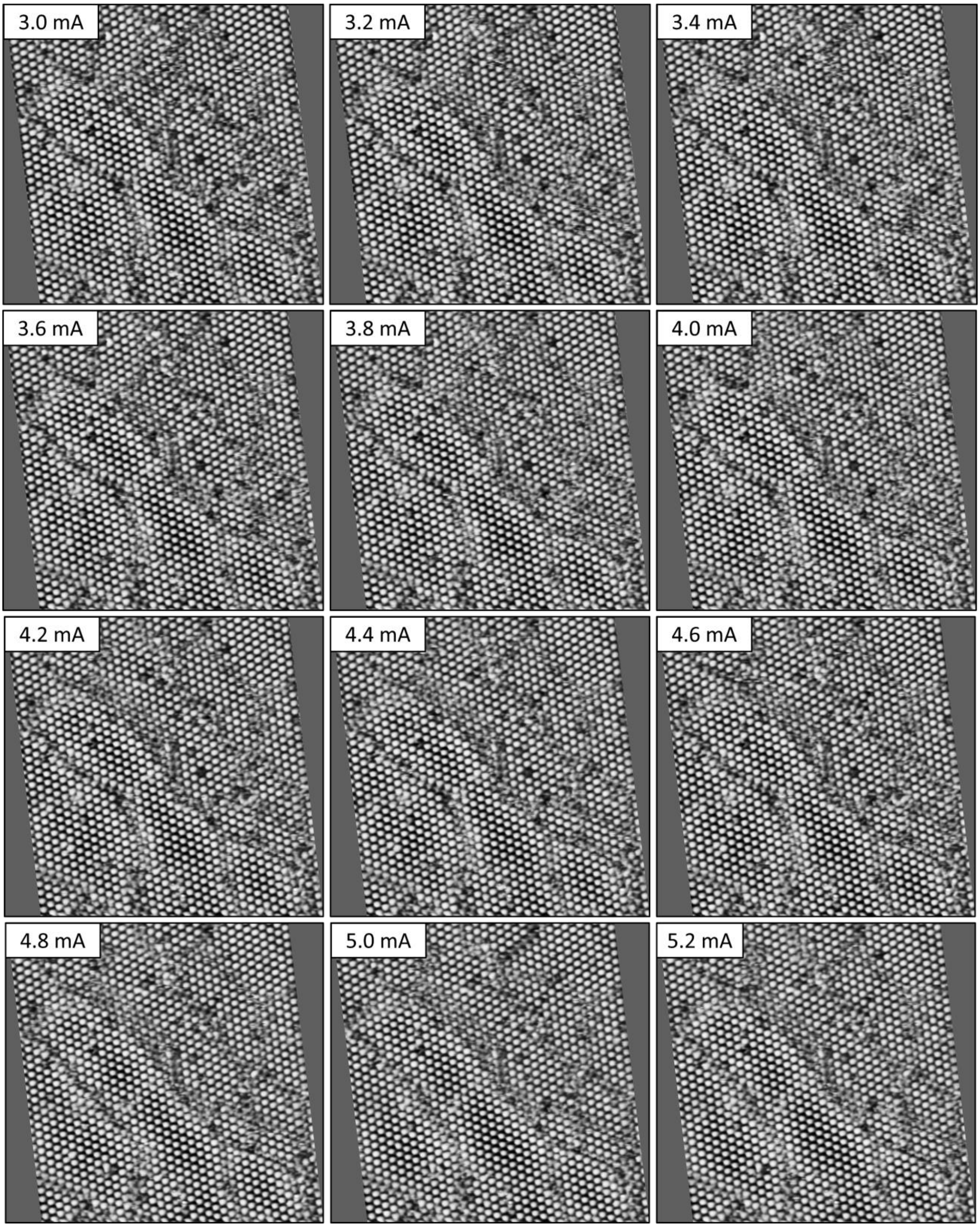

Supplementary Figure 7: Continuation of the repeated 'erase' sequence on a different 1T-TaS_2 sample at 4 K for current pulses from 3.0 to 5.2 mA.

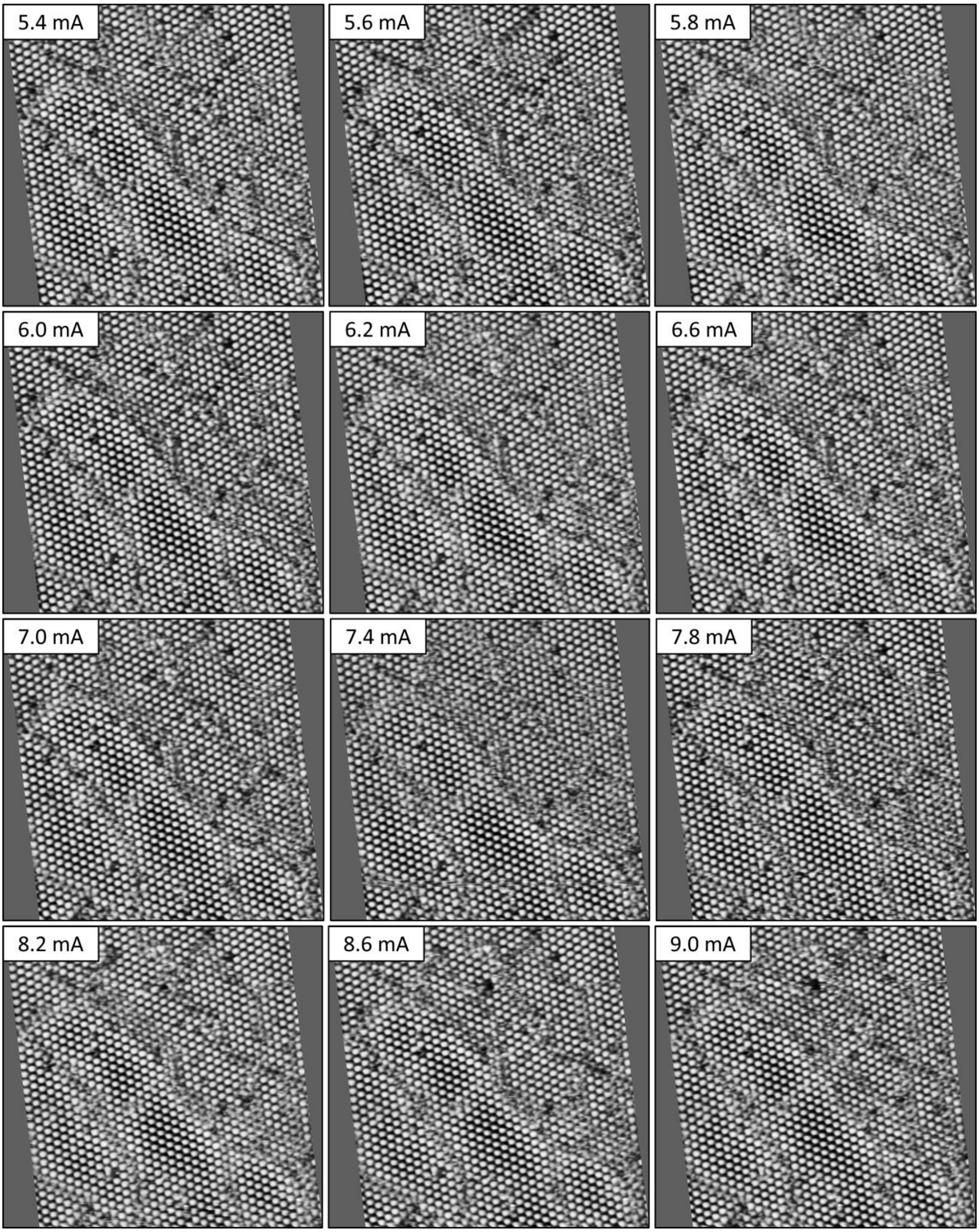

Supplementary Figure 8: Continuation of the repeated ‘erase’ sequence on a different 1T-TaS_2 sample at 4 K for current pulses from 5.4 to 9.0 mA.

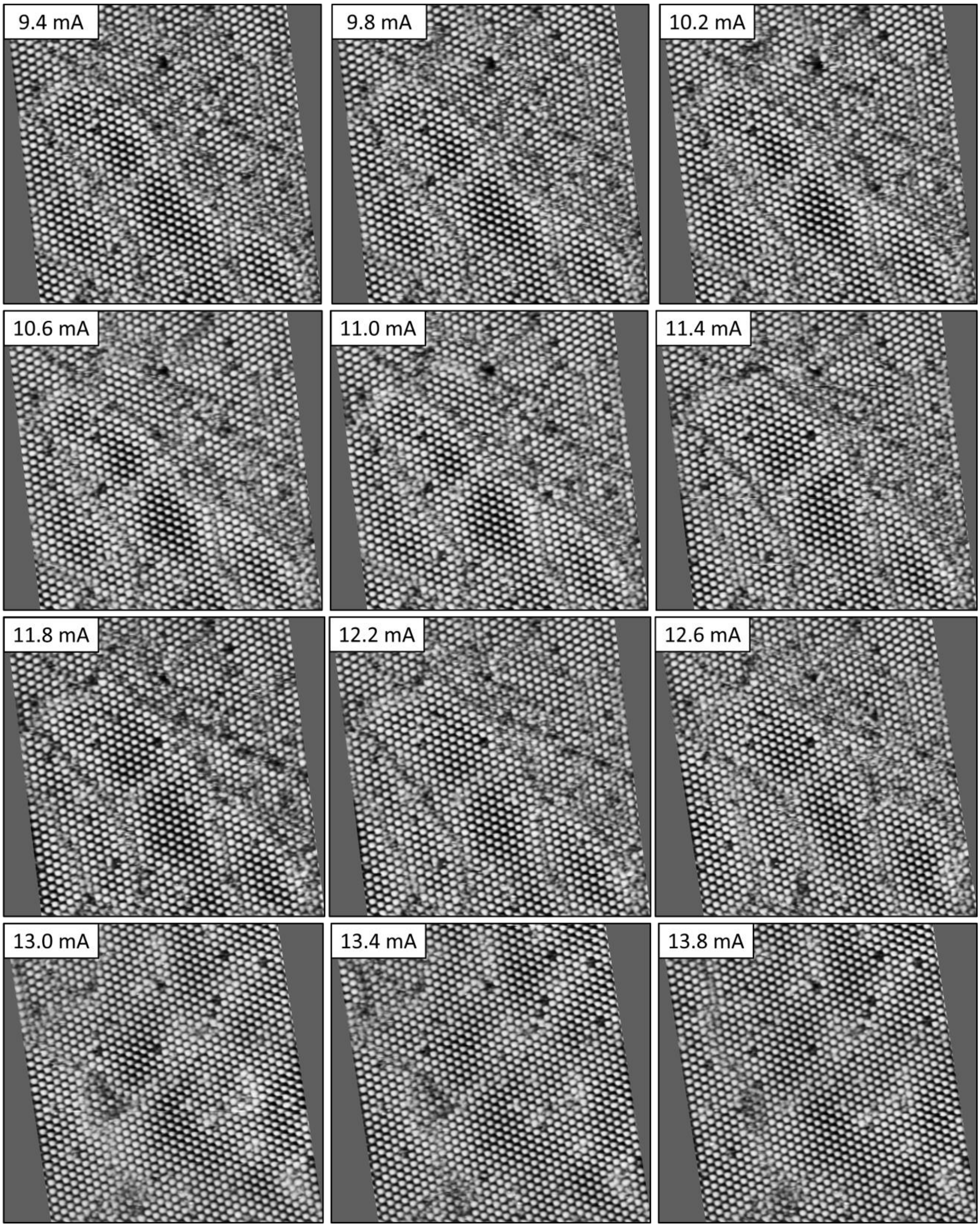

Supplementary Figure 9: Continuation of the repeated 'erase' sequence on a different 1T-TaS_2 sample at 4 K for current pulses from 9.4 to 13.8 mA.

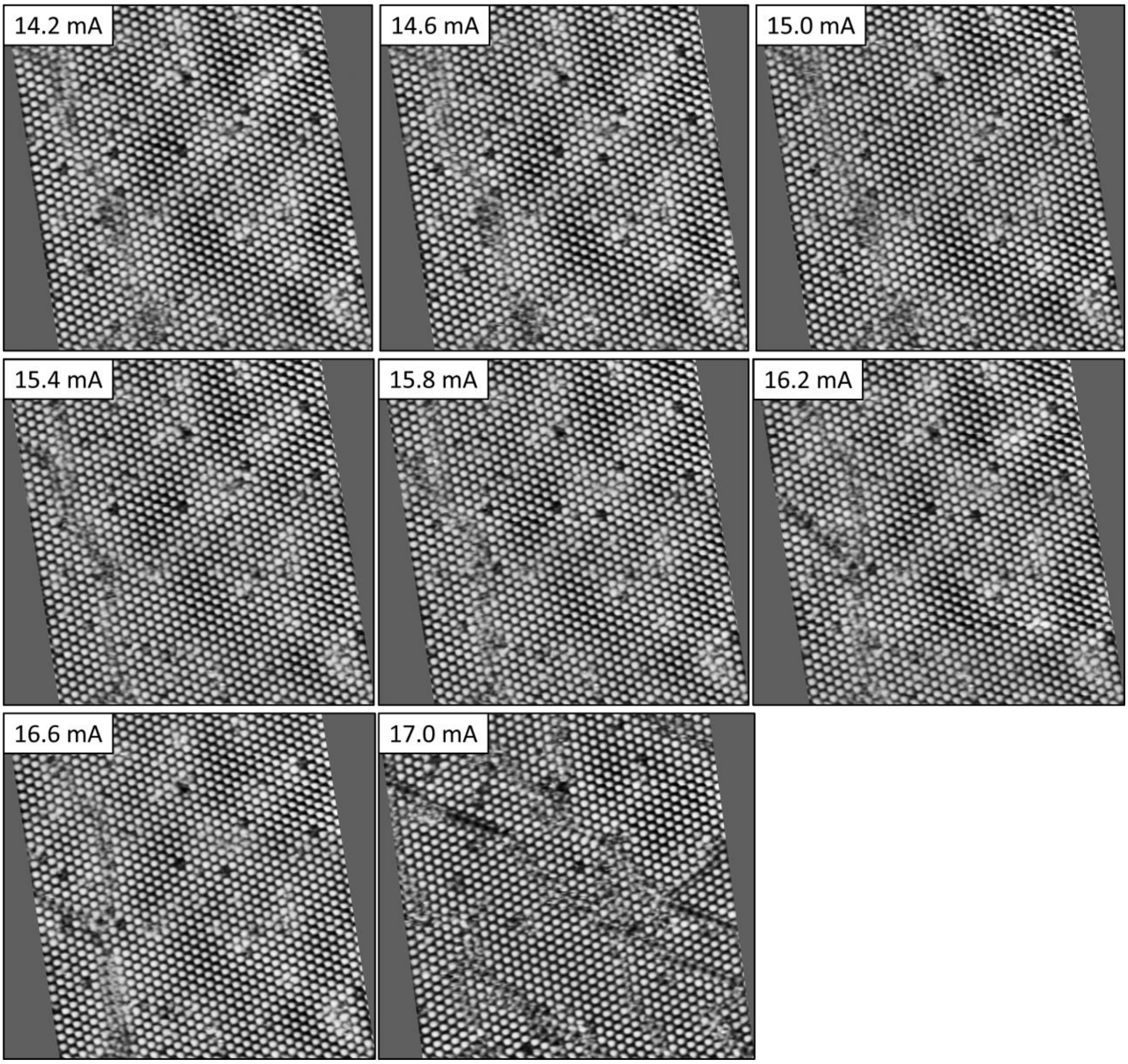

Supplementary Figure 10: Continuation of the repeated 'erase' sequence on a different 1T-TaS_2 sample at 4 K for current pulses from 14.2 to 17.0 mA. Last two panels (16.6 mA and 17.0 mA) show the substantially 'erased' surface of the sample and a 're-written' state, respectively.

Supplementary Note 5. STM MEASUREMENT OF THE SPATIAL CHARGE DENSITY DISTRIBUTION (COUNTING POLARONS)

A simplified expression for tunneling current $I(V)$ when a bias voltage V is applied between the tip and the sample is given by Supplementary Eq. (1):

$$I(V) = \int_{-\infty}^{\infty} P(E)\big(f(E-eV) - f(E)\big)dE \tag{1}$$

where $f(E)$is the Fermi-Dirac distribution and E is the energy. For typical STM measurements, where the tunneling is primarily between states near the Fermi level, the tunneling probability $P(E)$ is proportional to the product of the local density of states (LDOS) per unit volume of the sample

$N_{sample}(E)$ and the tip $N_{tip}(E)$ and the matrix element for tunneling $M_{i,f}$ between the initial and final states i and f: $P(E) \propto N_{tip}(E)\, N_{sample}(E) M_{i,f}$.

At low temperatures ($k_B T \ll E, V$) we can approximate $f(E) \sim 1$. Ignoring matrix element effects, and assuming that the tip is a simple broad band metal material such as tungsten or platinum, with $N_{tip}(E) \simeq constant$, the formula reduces to $I(V) \propto \int_{-\infty}^{\infty} N_{sample}(E) dE$. In a single band material, where W is the bandwidth of the state that crosses the Fermi level, the limits of integration can be replaced, such that $I(V) \propto \int_{-W/2}^{+W/2} N_{sample}(E) dE$.

In a polaronic Wigner crystal (or CDW) material, the LDOS is dependent on position due to the presence of localized electronic charges. The presence of charge can be detected by measuring the LDOS, and the tunneling current $I(\boldsymbol{r})$ effectively maps the spatial variation of the charge density. In the *C* state of 1T-TaS_2, the polaron is formed due to self-trapping and localization of an electron on the central Ta atom at the center of the Wigner-Seitz (WS) cell (Fig. 1a, b in main text). Its presence (or absence) is detected by STM tunneling. In the domain walls and vertices, the charge is no longer an integer, but can be fractional, as shown by our present analysis.

Experimentally, we observe that topographic images unambiguously reveal the presence or absence of polarons. For $V > \Delta$ (V=0.8 V), were $\Delta \simeq 0.3\ eV$ is the Mott gap, the polarons appear as symmetric objects with an electron on the Ta atom at the center of the David star (Fig. 1c and d). In Fig. 1b we show the topographic image revealing the sub-gap spatial structure of the atomic orbitals associated with the polaron for $V < \Delta$ (V=0.2 V). Presently we are not interested in the detailed electronic structure within the polarons, only in their presence (or absence). For a detailed discussion of the electronic structure within domain walls in 1T-TaS_2 from a microscopic viewpoint and high-resolution STM the reader is referred to the paper by Park et al[10].

Supplementary Note 6. The Wigner crystal limit and other theoretical approaches

While long-range-ordered, periodic discommensurate patchwork of the *NC* domain state can be described in terms of harmonics using conventional charge density wave (CDW) Landau theory[2,11], the intricate non-periodic features and topological features of the non-equilibrium states cannot. While various domain wall configurations may be set up for density-functional theory (DFT) modelling that gives quite good predictions of the local electronic structure[10], the approach does not address the origin of the domain structure, its dynamics or most importantly, the topological aspects of the nonequilibrium states. For this, the Wigner crystal approach has been shown to be much more appropriate.

The low carrier density in the *C* state in 1T-TaS_2 (1 electron per 13 unit cells) and relatively strong coupling of electrons with the lattice[3] lead to strong polaronic effects, and an enhanced carrier effective

mass. The material also exhibits plausible Fermi surface nesting[3], and a clear Kohn anomaly[12,13], which testifies to the strong electron-phonon interaction. Polaronic effects renormalize the electronic bands, which become narrow, resulting in an increase of the effective mass ($m* = 3 \sim 5$)[14]. The ratio of Coulomb to kinetic energy, commonly expressed in terms of the dimensionless ratio $r_s = \frac{V}{t} \simeq 70 \sim 100$ places the system in the Wigner crystal limit at low temperatures[15,16]. Altogether, the large r_s leads to a strong propensity of the electrons to localization[17]. The result is that the system may be thought of in terms of a 'polaronic' Wigner crystal state[16] in which the electrons are sparsely packed into an electronic crystal lattice[17] with one electron localized on every 13th Ta metal site. The unit cell $\sqrt{13}a \times \sqrt{13}a$, where a is the lattice constant, is shown in Fig. 1a. The localized electrons cause a polaronic lattice distortion in the form of a David star, one star per Wigner-Seitz (WS) primitive cell of the *C* superlattice (Fig. 1a). Fig. 1b shows a corresponding atomic resolution STM image of the surface of a 1T-TaS_2 crystal in the *C* state at 4 K. The Wigner crystal approach is justified by the intricate, aperiodic local real-space domain structures commonly detected by STM in this and related materials[17], which cannot be understood in terms of conventional Bloch theory.

We studied the interaction aspect of the Wigner crystal approach by simulating a charge lattice gas (CLG) model Hamiltonian $H = \sum_{i,j} V_{i,j} n_i n_j$, where n_i is the occupation number on a triangular atomic lattice site i, $V_{i,j} = V_0 \exp\left(-\frac{r_{i,j}}{r_0}\right)/r_{i,j}$ is the Yukawa screening potential with magnitude V_0 and screening radius $r_0 = 4.5\ a$, where $r_{i,j} = |\boldsymbol{r}_i - \boldsymbol{r}_j|$, $\boldsymbol{r}_i$ is the position of lattice site i and a is the lattice spacing. This CLG model exhibits an ordering transition from a high temperature liquid to a low temperature crystalline phase at $\frac{V_0}{k_B T} \approx 100$. We performed Metropolis Monte Carlo (MC) simulations using the simulated annealing (SA) technique, shown in Fig. 4 in main text, as well as parallel tempering (PT), shown here in SI, interchangeably. For both, the interaction was cut off to 0 for distances larger than 24 a, periodic boundary conditions were imposed and the number of polarons in the system was fixed throughout the simulation.

For SA we initialized a triangular lattice with 91×91 sites and $91 \times 91/13 + N$ polarons, where N is shown in Fig. 4, at the phase transition temperature and in an initial polaronic configuration such that all polarons are in one corner of the system. Afterwards, we decreased T in 100 equal steps to $\frac{V_0}{k_B T} = 1000$, where 1000 MC sweeps were performed at each step. Within 1 sweep, 91×91 attempts were made according to the Metropolis algorithm of moving 1 randomly chosen polaron 1 lattice site away from its current position.

In order to study the nature of emergent dislocations we also performed PT simulations with 32 replicas, where 3000 replica exchanges were attempted according to the Metropolis algorithm. The temperatures of replicas ranged from $\frac{V_0}{k_B T} = 10^2$ to $\frac{V_0}{k_B T} = 10^5$ in 32 equal steps. The simulation

between replica exchanges was the same as described above, but with 10 MC sweeps. The size of the lattice was set to 156×156 and the number of polarons was fixed to $156 \times 156/13 + 1 = 1873$. The purpose of the PT simulations was to check whether a global H state with $\sum_i B_i \neq 0$, surrounded by C state, is possible. Therefore, we fixed the movement of polarons along the edge of the system within a band of 10 atomic lattice sites wide and arranged them in a C state pattern ($1/13$ polaronic lattice). The rest of the polarons were initially put in the center one next to another which were then allowed to move during the simulation as shown in Supplementary Fig. 11.

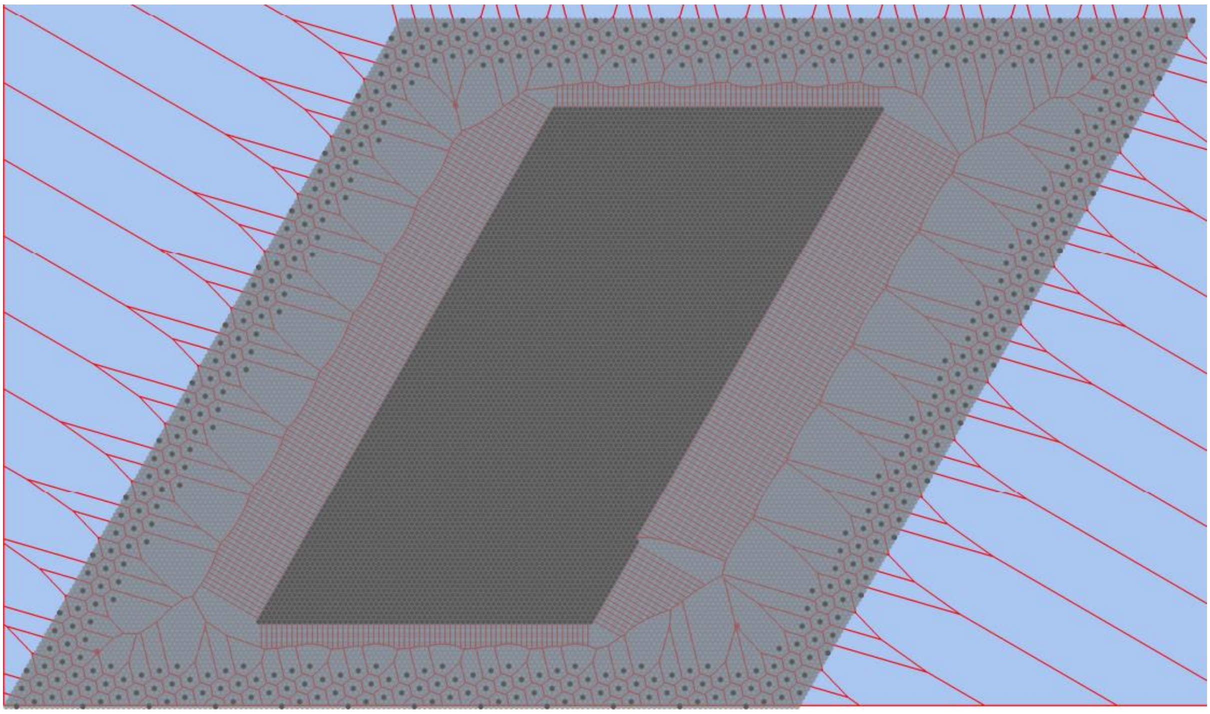

Supplementary Figure 11: Illustration of the initial polaronic configuration used in parallel tempering simulations. Polarons along the edge are not allowed to move, while polarons in the middle are. This figure shows too many polarons in the middle. In the actual simulation 1873 were used all together.

During the simulation, the polarons which are allowed to move, accommodate themselves firstly along the imposed fixed edge and then form some geometrically frustrated state in the middle. As is shown in Supplementary Fig. 12, we can clearly see that it is possible for the polarons to arrange in a pattern where $\sum_i B_i \neq 0$ globally. The authors believe that if the system was equilibrated even more carefully, the polaronic configuration would relax further in energy and cover a larger surface with the $1/13$ lattice, however this is out of the scope of this paper.

Supplementary Fig. 12a and b show a WS dislocation map and density map, respectively, where three patches of H state surrounded with C state can be determined. If Burger vectors are drawn for dislocations in all three patches, we can see that in one of the patches the sum of Burger vectors is zero (blue), while in the other two (black, red) that is not the case.

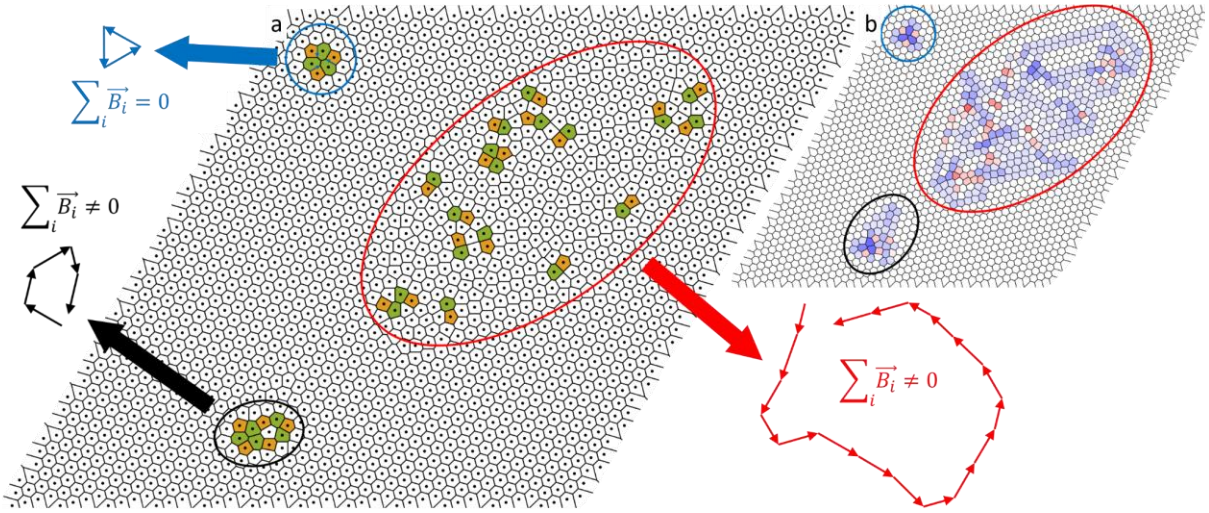

Supplementary Figure 12: a) Dislocation map of a simulated emergent state from a parallel tempering simulation, where three distinct patches of H state (blue, black, red) can be determined. Each patch is marked and the Burger vectors of each dislocation in the patch are drawn next to the image and summed. In one case the $\sum_i \boldsymbol{B_i} = \boldsymbol{0}$ (blue), while in two other cases $\sum_i \boldsymbol{B_i} \neq \boldsymbol{0}$ (black, red). b) Density map of the same simulation where individual patches can be more clearly distinguished.

Supplementary Note 7. FRACTIONAL SPATIALLY EXTENDED CHARGE

Here we focus on an example of a simulation with one injected electron (N=1) into the domain wall structure, presented via a charge density and a dislocation map in Supplementary Fig. 13a and b, respectively. We see how the added charge is distributed across the local patch composed of WS polygons with various surface areas (i.e. charge densities), where the area of the individual WS cell A_i is some fraction of the reference commensurate cell area A_C, and may be larger or smaller than A_C. For a finite number of injected charges N, the number of fractional WS polygons is finite, and so is their fractional surface area (i.e. charge density) because it is defined by the geometric construction in regards to the atomic lattice. Hence, even though there is a large number of potential fractional charge densities, fractional charge cannot be tuned continuously, but must follow the geometric constraints of the lattice. We note that certain fractions do appear more often than others. In Supplementary Fig. 13a we show the excess fractional charge density of each WS cell in regards to the surrounding reference C superlattice for the case of a single injected electron (N=1). By summing up individual fractions we get the total number of injected charges, in this case N=1 (with a slight rounding error), Supplementary Eq. (2). Note that the distortion in the WS lattice due to an injected electron extends beyond the dislocations, as seen by comparing the charge density and dislocation maps. The number of sides of individual WS cells may deviate from 6 (typical for C state), forming dislocations composed of 5-gon/7-gon or 4-gon/8-gon pairs.

$$\frac{\left(\frac{13}{10}+\frac{3611}{5000}-\frac{223}{500}+\frac{699}{500}-\frac{23}{40}+\frac{3611}{5000}+\frac{15111}{5000}+\frac{10063}{10000}+\frac{12139}{5000}+\frac{13}{10}+\frac{13}{10}+\frac{1}{10}+\frac{3611}{5000}\right)}{13}=1 \tag{2}$$

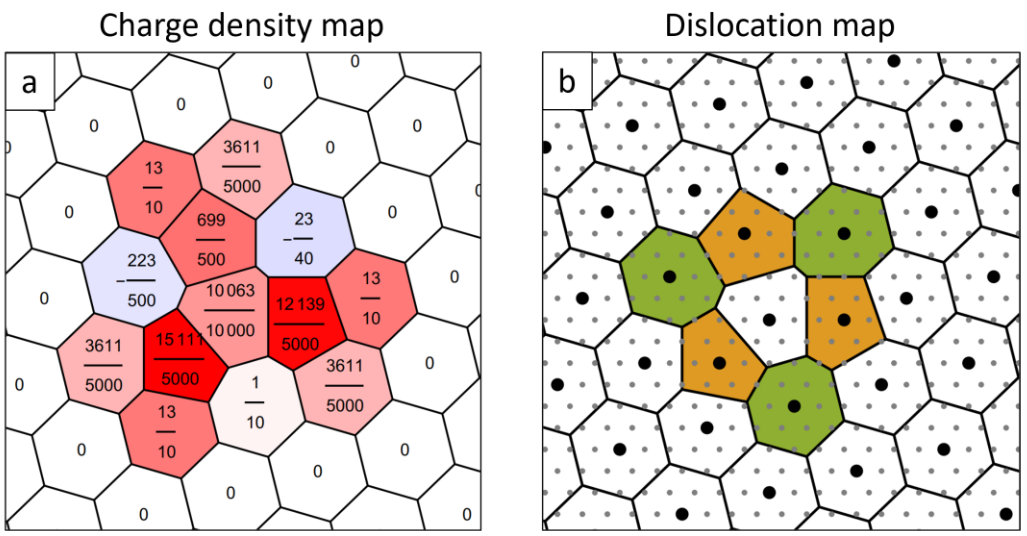

Supplementary Figure 13: Figure shows a distortion in the WS polaronic lattice in the case of a single injected electron (N=1) based on the CLG model MC calculation. a) Charge density map showing excess charge density of individual WS cells in relation to the surrounding C state. b) Dislocation map showing three pairs of 5-gon/7-gon dislocations in the WS polaronic lattice.

Supplementary Note 8. DISLOCATION ANALYSIS PROCEDURE

To be able to accurately produce the Wigner-Seitz (WS) construction on the acquired STM images, and therefore analyse the topology of the emergent domain structure in Fig. 3, detailed fitting of the STM images is required. The first step is fitting of the domains of polarons to negate the effect of drifting, and then determining the relative shift of each domain as seen in Supplementary Fig. 14a, using an algorithm already explained in ref.[18]. The colour code of each domain represents the displacement vector according to the insert to Supplementary Fig. 14a. In the second step only the centres of polarons are extracted, which are globally fitted to the underlying lattice, seen in Supplementary Fig. 14b. In the following step the missing polarons in the domain walls are inserted back according to the original STM image and the underlying lattice (Supplementary Fig. 14c). Once all the polaron positions are accurately determined, the WS construction is performed giving the end result in Supplementary Fig. 14d, where dislocations are clearly visible as pairs of 5-gons (brown) and 7-gons (green). Rest of the image consists of C superlattice domains of polarons (equilateral white 6-gons) and domain walls (deformed white 6-gons). This example shows the analysis procedure for Fig. 2, panel 4, and all other WS tessellations in Fig. 3b were performed in this manner.

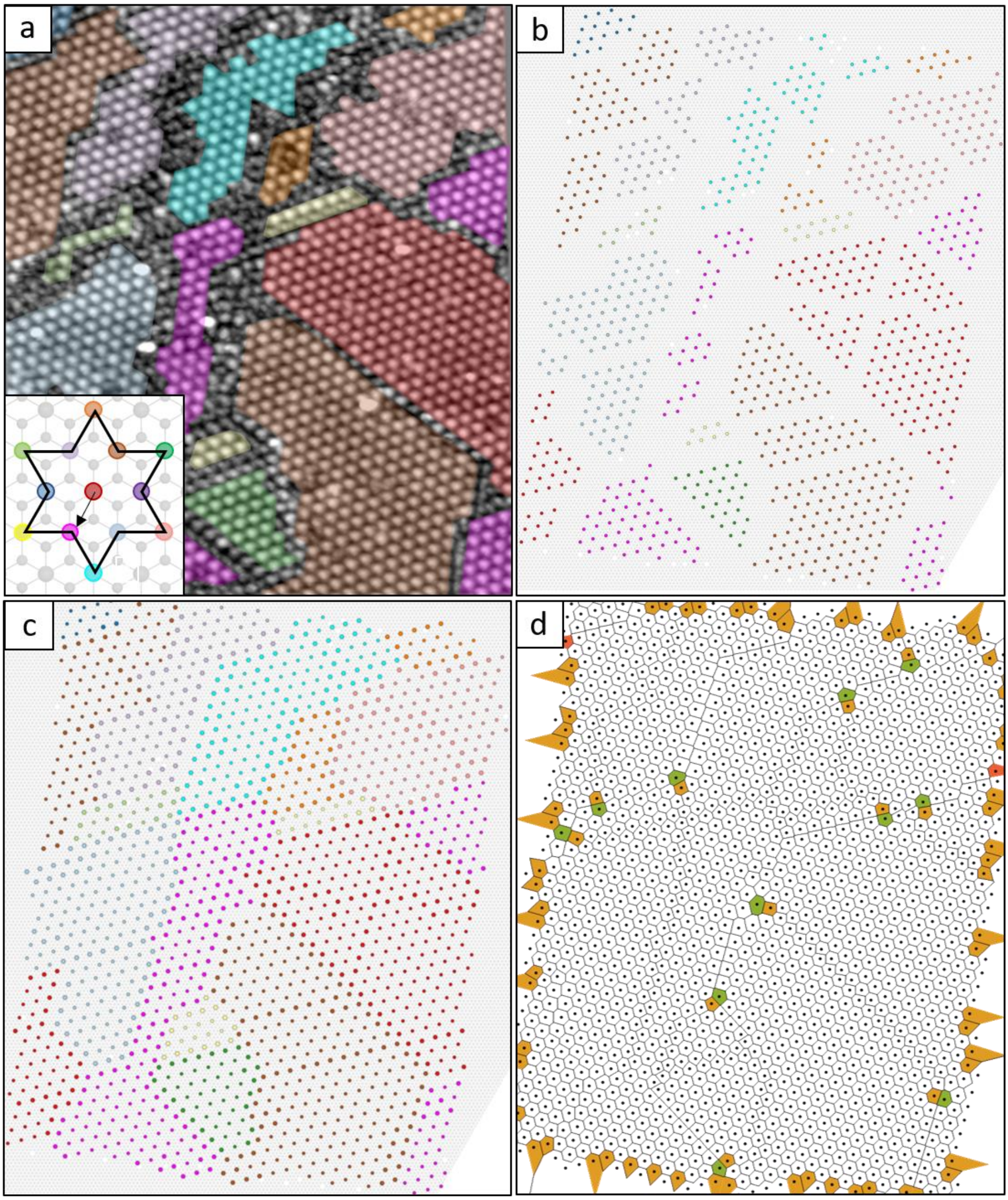

Supplementary Figure 14: a) STM image of H state with coloured domains of polarons that mark their displacement in accordance to the colour map in the insert. b) Centres of polarons extracted from Fig. 3a. c) Domain walls between domains are filled in with polarons according to the original STM image and the underlying lattice. d) WS tessellation performed on Fig. 3c. Dislocations are seen as pairs of 5-gons (brown) and 7-gons (green).

Supplementary Note 9. MODELLING THE TIP HEATING

Temperature- and state-dependent voltage-current (V-I) characteristics were simulated using the COMSOL Multiphysics software, which uses the finite element method (FEM) to solve partial differential equations governing heat conduction in solid materials (Supplementary Eq. (3) and (4)).

$$\rho C_p \frac{\partial T}{\partial t} - \nabla \cdot (k \nabla T) = Q_e \tag{3}$$

$$Q_e = \sigma |\nabla V|^2 \tag{4}$$

The thermal model was coupled to the electromagnetic model which provided the heat source term Q_e in the differential equations by calculating resistive losses in the device (Joule heating). The density ρ, heat capacity C_p [19], thermal conductivity k_{th} [20], and electrical conductivity σ of all the materials were taken from literature and/or experimentally calibrated[5,21].

The realistic geometry of the characterized system was imitated in the model as follows (Fig. 2d): each of the two tungsten STM probes in contact with the sample was represented as a cylinder measuring 6 μm in diameter. At the sample side, the cylinder turned into a 10 μm long cone which narrowed to a spherical tip measuring 40 nm in diameter. Both probes were assumed to be penetrating into the underlying 500 μm thick 1T-TaS_2 sample by 40 nm, while the lateral spacing between them was set to 2 μm. Surface contact resistance of $0.5 \times 10^{-12}\ \Omega\ \mathrm{m}^2$ was applied to the interface between the STM probes and the 1T-TaS_2 substrate. Thermal contact with the cryostat cold finger at 4 K was traced via the bottom of the substrate and also via both STM probes.

In simulations we assumed that prior to excitation, at 4 K, the entire 1T-TaS_2 sample was initially either in the HI (*C*) state or in a mixture of HI (*C*) and LO (*H*) states (the latter case representing situation immediately following a 'write' process). For exact calibration of the model in each initial state, we used measured R-T curves shown in Supplementary Fig. 1c. We then applied current excitation of a gradually increasing magnitude through the STM probes. The corresponding V-I characteristics and the temperature between the probes at the surface of 1T-TaS_2 were extracted from simulation results, as presented in Fig. 2d.

By studying the simulated V-I curves, it can be observed that below 1 mA the behaviour in the *C* state is close to the measured data (red triangles), but shows saturation at somewhat higher currents than in the experiment. Similar behaviour is observed in the transition from the *H* to the *C* state, with a similar departure at high currents. In the calculation, a strong nonlinearity appears near 2mA, which arises from the $H \rightarrow C$ transition between 40 ~ 60 K (Fig. 2d). A nonlinearity is also noticeable in the experiment, but is not as pronounced. Part of the discrepancy between the model and the data may be due to the fact that the R-T curve used in the calibration of the model was measured with conventional parallel contact electrodes, which is different to the experiment where point contact was used (STM tips). Another reason for the discrepancy may be attributed to the fact that the measurement protocols are different

(see Methods). Nevertheless, considering that the calculations are based on measured thermal and electrical constants, and there are no adjustable parameters, the agreement between the data and the model for both curves is quite impressive, particularly the presence of the non-linearity near 2mA, arising from the $H \rightarrow C$ transition at $40\sim60$ K (Supplementary Fig. 1c).

REFERENCES

1. Wilson, J. A., Di Salvo, F. J. & Mahajan, S. Charge-density waves and superlattices in the metallic layered transition metal dichalcogenides. *Adv. Phys.* **24**, 117–201 (1975).
2. McMillan, W. L. Theory of discommensurations and the commensurate-incommensurate charge-density-wave phase transition. *Phys. Rev. B* **14**, 1496–1502 (1976).
3. Rossnagel, K. On the origin of charge-density waves in select layered transition-metal dichalcogenides. *J. Phys. Condens. Matter* **23**, 213001 (2011).
4. Tanda, S., Sambongi, T., Tani, T. & Tanaka, S. X-Ray Study of Charge Density Wave Structure in 1T-TaS $_2$. *J. Phys. Soc. Jpn.* **53**, 476–479 (1984).
5. Stojchevska, L. *et al.* Ultrafast Switching to a Stable Hidden Quantum State in an Electronic Crystal. *Science* **344**, 177–180 (2014).
6. Vaskivskyi, I. *et al.* Fast electronic resistance switching involving hidden charge density wave states. *Nat. Commun.* **7**, (2016).
7. Mraz, A. *et al.* Charge Configuration Memory Devices: Energy Efficiency and Switching Speed. *Nano Lett.* **22**, 4814–4821 (2022).
8. Gerasimenko, Y. A. *et al.* Quantum jamming transition to a correlated electron glass in 1T-TaS2. *Nat. Mater.* **18**, 1078–1083 (2019).
9. Vaskivskyi, I. *et al.* Controlling the metal-to-insulator relaxation of the metastable hidden quantum state in 1T-TaS $_2$. *Sci. Adv.* **1**, e1500168 (2015).
10. Park, J. W., Lee, J. & Yeom, H. W. Zoology of domain walls in quasi-2D correlated charge density wave of 1T-TaS2. *Npj Quantum Mater.* **6**, 32 (2021).
11. Nakanishi, K. & Shiba, H. Domain-like Incommensurate Charge-Density-Wave States and the First-Order Incommensurate-Commensurate Transitions in Layered Tantalum Dichalcogenides. I. 1T-Polytype. *J. Phys. Soc. Jpn.* **43**, 1839–1847 (1977).
12. Ziebeck, K. R. A., Dorner, B., Stirling, W. G. & Schollhorn, R. Kohn anomaly in the 1T $_2$ phase of TaS $_2$. *J. Phys. F Met. Phys.* **7**, 1139–1143 (1977).

13. Machida, Y. *et al.* Observation of Soft Phonon Modes in 1 *T* -TaS $_2$ by means of X-ray Thermal Diffuse Scattering. *J. Phys. Soc. Jpn.* **73**, 3064–3069 (2004).

14. Rossnagel, K. & Smith, N. V. Spin-orbit coupling in the band structure of reconstructed 1 T − TaS 2. *Phys. Rev. B* **73**, 073106 (2006).

15. Kivelson, S., Kallin, C., Arovas, D. P. & Schrieffer, J. R. Cooperative ring exchange and the fractional quantum Hall effect. *Phys. Rev. B* **36**, 1620–1646 (1987).

16. Karpov, P. & Brazovskii, S. Modeling of networks and globules of charged domain walls observed in pump and pulse induced states. *Sci. Rep.* **8**, (2018).

17. Vodeb, J. *et al.* Configurational electronic states in layered transition metal dichalcogenides. *New J. Phys.* **21**, 083001 (2019).

18. Kranjec, A. *et al.* Electronic Dislocation Dynamics in Metastable Wigner Crystal States. *Symmetry* **14**, 926 (2022).

19. Suzuki, A., Koizumi, M. & Doyama, M. Thermal evidences for successive CDW phase transitions in 1T-TaS2. *Solid State Commun.* **53**, 201–203 (1985).

20. Núñez-Regueiro, M. D., Lopez-Castillo, J. M. & Ayache, C. Thermal Conductivity of 1 T -Ta S 2 and 2 H -Ta Se 2. *Phys. Rev. Lett.* **55**, 1931–1934 (1985).

21. Mihailovic, D. *et al.* Ultrafast non-thermal and thermal switching in charge configuration memory devices based on 1T-TaS $_2$. *Appl. Phys. Lett.* **119**, 013106 (2021).
