# Supplementary material for: Manipulation of fractionalized charge in the metastable topologically entangled state of a doped Wigner crystal
Source: Nat Commun. 2023 Dec 11;14:8214. doi: 10.1038/s41467-023-43800-3 (PMC10713626; doi:10.1038/s41467-023-43800-3)
Supplement: Supplementary file 3 — Description of Additional Supplementary Files [file 41467_2023_43800_MOESM3_ESM.docx]

**Description of Additional Supplementary Files**

**File Name: Supplementary Movie 1
Description:** Full set of STM images of the 'erase' sequence in Fig. 2 in the main manuscript.

**File Name: Supplementary Movie 2**

**Description:** The repeated ‘erase” sequence on a different 1T-TaS2 sample at 4 K.
